# Cumulus cell-mediated sperm selection enhances blastocyst quality using sibling oocytes

**DOI:** 10.1007/s10815-026-03818-0

**Published:** 2026-02-02

**Authors:** Jorge Ten, Nerea Díaz, Miguel Herreros, Ángel Máñez-Grau, José Antonio Ortiz, Adoración Rodríguez-Arnedo, Mónica Aparicio, Elisa Álvarez, José Luis Girela, Juan Carlos Castillo, Andrea Bernabeu

**Affiliations:** 1 Embryology Unit, Instituto Bernabeu, Alicante, Spain; 2 Biotechnology Department, University of Alicante, Alicante, Spain; 3 Biotech, Instituto Bernabeu, Alicante, Spain; 4 Reproductive Medicine, Instituto Bernabeu, Alicante, Spain; 5 Chair of Reproductive and Community Medicine, Miguel Hernandez University, Elche, Spain

**Keywords:** Sperm selection, Cumulus cells, ICSI, Embryo quality

## Abstract

**Purpose:**

Can sperm selection through cumulus cells improve embryo quality compared to conventional methods, and is its effectiveness influenced by parental age?

**Methods:**

This prospective clinical trial included 99 ICSI cycles from 95 couples. Sibling oocytes were randomly allocated at the oocyte level to either the study group (cumulus cell-mediated sperm selection after conventional density gradients centrifugation (DGC), 554 oocytes) or the control group (only sperm selection by DGC, 543 oocytes), using a dish designed to facilitate sperm interaction with cumulus cells. The inclusion criteria for this study were patients using their own oocytes, with a medical indication for ICSI, who had at least 6 mature oocytes (MII) in that cycle. For semen samples, inclusion required the ability to adjust the concentration to 10 million/mL. Exclusion criteria included the use of vitrified oocytes, donated oocytes, and semen samples obtained by testicular biopsy or aspiration. Embryo quality was assessed at the blastocyst stage on day 5 according to ASEBIR. A subanalysis evaluated the influence of parental age on outcomes.

**Results:**

The study group showed a significantly higher proportion of good-quality day-5 blastocysts compared to controls (55.2% vs. 45.3%, *p* = 0.028). No statistically significant differences were observed in overall blastocyst formation or pregnancy rates, although favourable trends were noted. In an age-stratified analysis, a significant improvement in day-5 blastocyst quality among evaluable blastocysts was observed in women aged 40–45 (51.4% vs. 30.4%, *p* = 0.017), with a non-significant trend toward improved outcomes in men aged 40–53 (44.7% vs. 32.6%, *p* = 0.083). No differences were seen in younger age groups.

**Conclusion:**

Cumulus cell-mediated sperm selection after DGC using a specialized Oosafe® ICSI Dish with Sperm Selection Channels was associated with an increased proportion of good-quality day 5 blastocysts compared with conventional sperm preparation. While clinical outcomes did not differ significantly, these findings suggest a potential benefit in specific ART subpopulations, particularly those of advanced maternal age. Further adequately powered studies are required to confirm these observations and to determine their impact on clinical outcomes.

## Introduction

Infertility affects approximately 17.5% of individuals worldwide and is recognized as a disease by the World Health Organization [1]. Increasing maternal and paternal ages are major determinants of reduced reproductive potential. Advanced maternal age is associated with impaired oocyte quality and embryo development [2], while advanced paternal age has been linked to altered sperm quality and compromised embryonic development [3].

Male factor infertility contributes to nearly 50% of infertility cases and is frequently associated with impaired sperm quality, including reduced motility, concentration, and DNA integrity [4]. Conventional semen analysis primarily evaluates macroscopic parameters such as concentration, motility, and morphology; however, increasing evidence indicates that molecular and physiological factors, particularly sperm DNA fragmentation (SDF), play a critical role in embryo development and assisted reproductive technology (ART) outcomes [5]. Elevated SDF levels have been associated with increased miscarriage rates and impaired embryo development, underscoring the importance of selecting sperm with high fertilization potential (Borges et al. 2019).

Sperm selection plays a critical role in ART, particularly during intracytoplasmic sperm injection (ICSI), where spermatozoa are chosen based on motility and morphology. However, routine selection techniques, such as density gradient centrifugation (DGC) and swim-up (SU), involve aggressive centrifugation steps that can increase sperm DNA damage due to elevated reactive oxygen species (ROS) generation [4]. Additionally, these methods require multiple steps of sample manipulation, increasing the risk of iatrogenic damage. This scenario has driven the development of alternative, more physiological sperm selection methods.

A promising approach involves the use of cumulus cells (CCs), granulosa cells that naturally surround the oocyte and play a vital role in supporting oocyte maturation and fertilization. CCs secrete molecules such as progesterone, prostaglandins, and hyaluronic acid, which promote key fertilization processes, including hyperactivation, the acrosome reaction, and tyrosine phosphorylation [6]. Moreover, the extracellular matrix of CCs acts as a natural selective barrier, favouring sperm with optimal motility, morphology, and DNA integrity [7]. CCs also facilitate bidirectional communication with the oocyte, promoting its maturation, regulating antioxidant production, and mitigating oxidative stress [8].

Research has demonstrated that sperm interacting with CCs exhibit improved fertilization potential, characterized by enhanced morphology, motility, and DNA integrity [9]. Notably, sperm selected through CCs display reduced levels of DNA fragmentation compared to those processed using conventional methods [10]. In addition, exposure to the CCs secretome has been shown to improve mitochondrial function and sperm motility [9], suggesting that CCs may act as natural filters, replicating the physiological sperm selection mechanisms of the female reproductive tract. These findings underscore the potential of CC-based selection strategies, particularly in the context of advanced paternal age. An observational study by Kaarouch et al. [11] reported significantly higher rates of DNA fragmentation, chromatin decondensation, and aneuploidy in sperm from older men compared to younger individuals. Taken together, these insights suggest that CC-mediated selection may offer a promising strategy to counteract age-related deterioration in sperm genomic quality. A pilot study previously reported [12] investigated the efficacy of sperm selection via CCs using a conventional ICSI dish. The findings demonstrated that this method may enhance embryo development and quality, underscoring its potential clinical value in improving ART outcomes. Building on these results, a study evaluated a novel CC-based sperm selection device [13] (Oosafe® ICSI Dish with Sperm Selection Channels; CE marking pending; patent publication number EP4303299). Their study demonstrated that this device significantly reduced sperm DNA fragmentation while maintaining motility and functional competence, strongly supporting its clinical application.

In this study, we further investigated the impact of sperm selection through CCs on embryo development outcomes compared to a control group using DGC. The specially designed Oosafe® ICSI Dish with Sperm Selection Channels device was utilized to standardize the selection process, ensuring reproducibility. Additionally, a subanalysis was conducted to evaluate whether maternal and paternal age had any influence on the observed outcomes. This analysis aimed to determine if the effect of sperm selection using this device varied across different age ranges, providing further insight into its potential benefits in specific patient subgroups.

## Materials and methods

### Study characteristics, population, and randomization

This prospective clinical trial was approved by the appropriate Ethical Committee for Drug Research and by an Internal Institutional Review Board. Eligible patients were informed about the study objectives and procedures, and those who provided signed informed consent were enrolled. No interventions deviating from standard clinical practice were performed prior to obtaining informed consent. The trial commenced in July 2022 and concluded in December 2024. This study included only patients with a medical indication for ICSI during their assisted reproduction cycle in the IVF laboratory and a BMI < 30 kg/m^2^. A total of 95 couples were enrolled, accounting for 99 treatment cycles and yielding 1097 mature oocytes. Of these, 543 oocytes were allocated to the control group and 554 to the intervention group. The mean age of female participants was 36.73 years in the intervention group and 36.69 years in the control group. Among male partners, the mean ages were 38.86 and 38.33 years in the intervention and control groups, respectively.

Eligibility criteria required patients to undergo ovarian stimulation using their own fresh oocytes, yielding at least six mature (MII) oocytes. Additionally, embryo development to the blastocyst stage was mandatory, and semen samples had to be obtained via ejaculation and had the ability to adjust the concentration to 10 million/mL.

Exclusion criteria included the use of vitrified oocytes, retrieval of fewer than six mature oocytes, or sperm collection through testicular biopsy or puncture and donor samples. Patients were also excluded if an insufficient number of sperm successfully traversed the cumulus barrier during the selection process or if the final sperm concentration did not reach 10 million/mL, in which case conventional ICSI was performed instead.

**Fig. 1:**
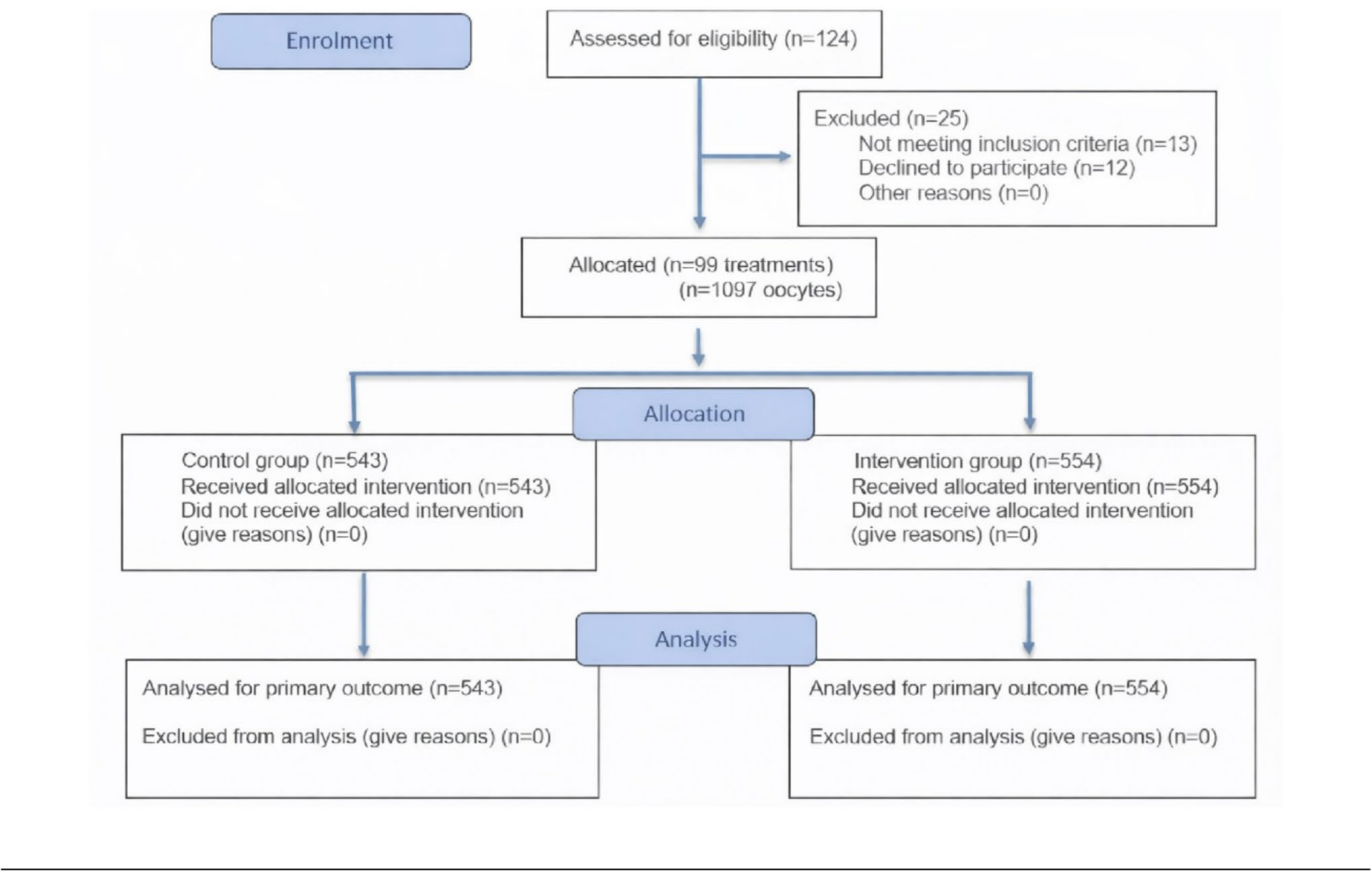
Flow diagram of the progress through the phases of the study showing oocyte-level allocation of sibling oocytes to the two study groups, adapted from [15]

The distribution of infertility etiology within the study population was categorized as follows. In the intervention group, the causes of infertility were classified as unexplained (11.0%), female factor (59.9%), male factor (11.7%), and combined factors (17.3%). In the control group, the corresponding proportions were 8.8% for unexplained infertility, 61.3% for female factor, 14.2% for male factor, and 15.7% for combined etiologies. Each oocyte was assigned to a study group using simple randomization, a method that ensures an equal probability of allocation to either group. This procedure minimizes selection bias and increases the likelihood that observed differences are attributable to the interventions rather than to pre-existing characteristics. Randomization was carried out using the!RNDSEQ macro of SPSS Statistics [14], so that both groups had the same probability of being assigned at each point in time. The enrolment details are represented in Fig. 1.

### Sample size calculation and study variables

The primary objective of this study was to assess the proportion of embryos resulting in a good-quality day-5 blastocyst (grades A and B) according to the criteria established by the Spanish Association for the Study of Reproductive Biology (ASEBIR) [16]. For the purposes of this study, good-quality blastocysts were defined as those considered suitable for embryo transfer and/or cryopreservation in routine clinical practice. Embryos were defined as fertilized oocytes presenting two pronuclei and two polar bodies (2PN + 2CP).

For the sample size calculation, we assumed a 45% proportion of good-quality blastocysts (among evaluable blastocysts) in the control group and anticipated an increase to 55% following cumulus cell-mediated sperm selection (10% absolute increase), based on results from a previous pilot study [12]. Under these assumptions, a total of 784 embryos (392 per group) were required to achieve 80% power with a two-sided *α* of 0.05. The corresponding estimate of 1288 oocytes was derived from assumptions regarding oocyte maturation, fertilization rates (70%), and losses during embryo culture (15%) and should therefore be considered an approximation. Across the 99 cycles included, a total of 1387 oocytes were retrieved, of which 1097 (79.1%) reached metaphase II (MII) and were eligible for allocation. These 1097 mature oocytes were randomly allocated at the oocyte level within each cycle by simple randomization, yielding 839 fertilized oocytes (2PN + 2 PB) available for embryo developmental assessment. Secondary variables encompassed fertilization rate, blastocyst formation rate, positive pregnancy rate, and the ongoing pregnancy rate, defined as pregnancies progressing beyond 12 weeks of gestation. The pregnancy test (β-hCG level) was assessed in blood samples taken 13 days post-ICSI in fresh transfers and 9 days post transfer in cryotransfers. According to routine clinical policy, embryo transfer is performed as single blastocyst transfer (SET), and double embryo transfer is not contemplated. Accordingly, all embryo transfers included in this study were single blastocyst transfers. This policy ensured unequivocal attribution of clinical outcomes to a specific embryo and its corresponding sperm selection group.

Additional parameters such as maternal and paternal age, ovarian stimulation protocol, duration of stimulation, type of ovulation trigger, infertility etiology, and semen sample quality were also recorded. A statistical subanalysis is included to assess the impact of maternal and paternal age on these primary and secondary outcomes.

### Ovarian stimulation

The controlled ovarian stimulation (COS) protocol was personalized by a fertility specialist, who selected the type, dosage, and duration of gonadotropin treatment based on each patient’s clinical profile, BMI, and ovarian reserve markers like antral follicle count and AMH levels. Protocols were adjusted according to ovarian response, and some patients used oral contraceptives in the months prior to stimulation.

Stimulation protocols vary by hormone type and cycle phase at treatment initiation. Mild protocols use lower gonadotropin doses, sometimes combined with oral agents. Final oocyte maturation was triggered with either hCG or, in antagonist protocols, a GnRH agonist to stimulate endogenous LH and FSH.

Follicular development was monitored via transvaginal ultrasound. When at least two follicles reached ≥ 18 mm, the trigger was administered. Oocyte retrieval occurred 36 h later under sedation via ultrasound-guided transvaginal aspiration.

### Development and structure of the ICSI dish

**Fig. 2:**
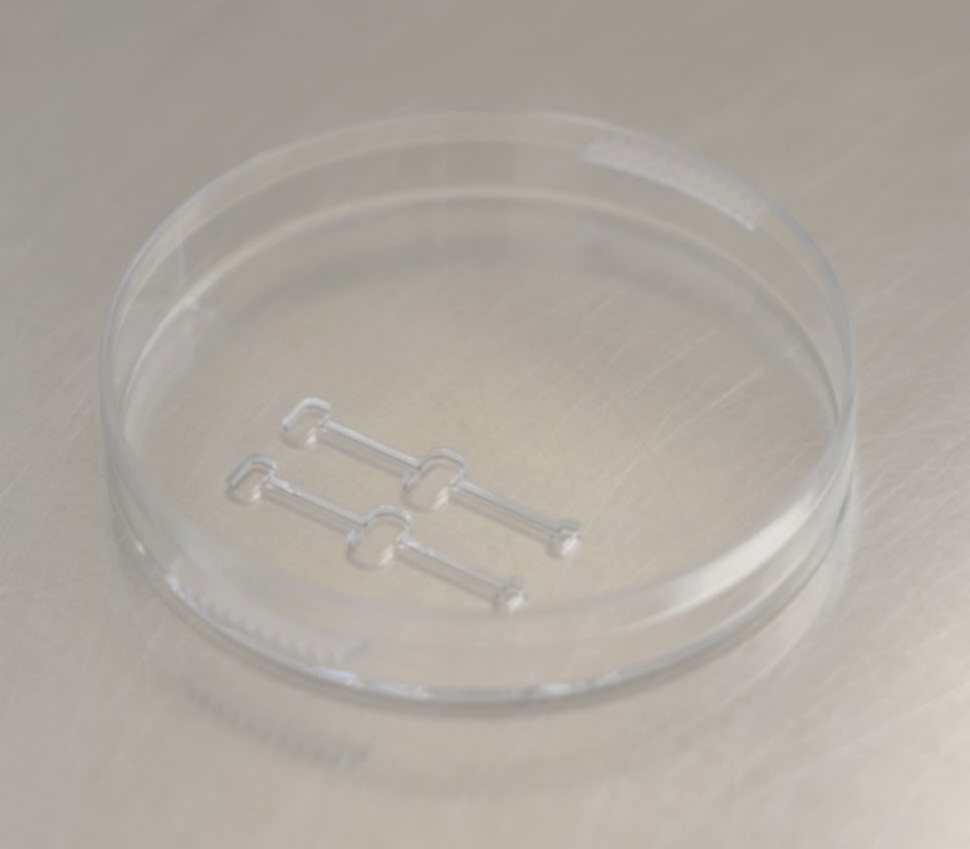
Oosafe® ICSI dish with sperm selection channels

**Fig. 3:**
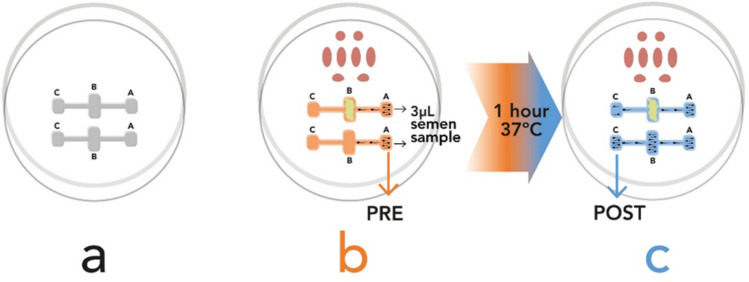
Dish functioning. **a** ICSI dish with the 3 wells (A, B, and C). **b** Upper lane with CCs in the B well and bottom lane without CCs, semen samples (PRE samples) are deposited in Well A. **c** After 1 h incubation at 37 °C, spermatozoa are collected from Well C (POST-samples)

The sperm selection device utilized in this study was an ICSI dish with two channels (*Oosafe® ICSI Dish with Sperm Selection Channels*, CE mark pending, patent publication number EP4303299, Fig. 2), a specialized tool designed to enhance sperm selection for assisted reproduction. The device used was evaluated and approved by an Ethical Committee for Drug Research and by an Institutional Review Board and is manufactured using embryo-tested materials under MD Regulation (EU) 2017/745. The product is made in ISO 13485:2016 & ISO 9001:2015 certified facility. Every batch undergoes Gamma sterilization and is tested for Mouse Embryo Assay, Endotoxin (LAL), and Human Sperm Survival and motility after manufacturing. This dish integrates two main compartments to streamline sperm selection and intracytoplasmic sperm injection (ICSI): an upper section dedicated to the ICSI procedure and a lower section consisting of two parallel lanes (Fig. 3).

Each lane runs longitudinally across the dish and is divided into three distinct wells, labelled A, B, and C:

- Well A: Located at the right end of each lane, Well A is used for the deposition of processed semen samples prepared via density gradient centrifugation.
- Well B: Positioned in the center of each lane, Well B is designed for the placement of cumulus cells freshly harvested previous oocyte denudation. In this study, cumulus cells were not included in the channel of the control group.
- Well C: Found at the left end of each lane, Well C collects spermatozoa that successfully traverse the cumulus cells placed in Well B. The selected spermatozoa in Well C are considered optimal candidates for subsequent fertilization via microinjection.

A notable feature of the dish is the presence of one lane that serves as a control, devoid of cumulus cells. This control lane enables a direct comparison of sperm motility and selection in the presence versus absence of cumulus cells (Fig. 3b).

The upper compartment of the dish facilitates the ICSI process, allowing for a seamless workflow. Selected spermatozoa from Well C can be directly microinjected into oocytes within the same dish, minimizing handling and preserving sample integrity.

This precise configuration ensures a standardized and efficient sperm selection process, providing a reproducible platform for advanced assisted reproduction protocols.

### Preparation of seminal sample

Semen samples were collected in sterile containers on the day of follicular puncture after a 2–3 days period of sexual abstinence, obtained through masturbation. Upon liquefaction at room temperature, each semen sample underwent analysis in a Makler chamber to evaluate sperm concentration and motility. Subsequently, all samples included in the study underwent DGC. Following completion of the gradient, sperm concentration was adjusted to 10 × 10^6^ spermatozoa/mL.

### Preparation of cumulus cells

Following follicular puncture, Cumulus-Oocyte Complex (COCs) were incubated at 37 °C in Global Total® for fertilization culture medium (LifeGlobal, CooperSurgical, Denmark) under low oxygen pressure until the decumulation process. COCs exhibiting normal morphology were selected for the study. A high-quality COC was defined as one presenting an expanded and loosely organized cumulus mass, indicative of appropriate oocyte maturation. Specifically, COCs displaying a compact cumulus structure or the presence of blood clots were excluded [17]. Between two and four COCs were selected per patient, depending on their size and morphological quality. Before extracting cumulus cells, the complexes were transferred to a 60-mm Petri dish filled with Global Total with HEPES® medium (LifeGlobal, CooperSurgical, Denmark) heated in an incubator at 37.0 °C for 30 min before use. Mechanical extraction of cumulus cells was performed using needles coupled to syringes, with a portion of the cumulus cells removed and stored in Global Total with HEPES® medium for subsequent dish preparation. Despite mechanical dissection, the structural and functional integrity of the cumulus cells was preserved, maintaining their extracellular matrix architecture until the moment they were penetrated by spermatozoa.

### Sperm selection on the dish

**Fig. 4:**
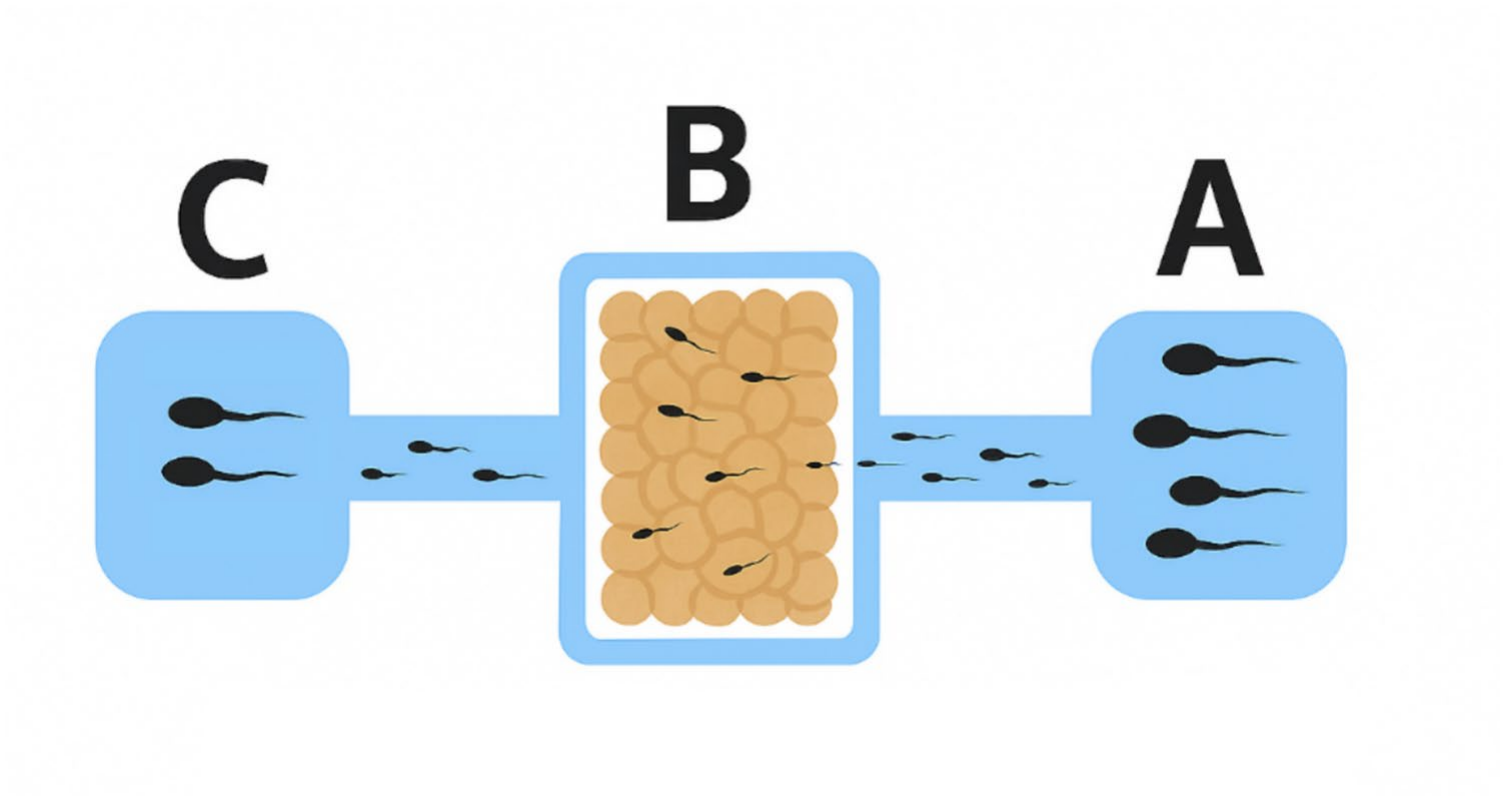
Enlarged view of the lane corresponding to the study group. *A* Semen sample added. *B* Sperm is passing through the cumulus cells. *C* After 1 h, spermatozoa are ready for collection and subsequent microinjection

First, lanes of the dish were filled with 10 µL of the same buffer medium, Sperm Washing medium® (Irvine Scientific, Fujifilm, Japan), with which the semen sample had been processed. Next, cumulus cells were deposited, completely filling the widened central part of the lanes (Well B, only in the study group, Fig. 4) and then, 3 µL of previously adjusted to 10 mill/mL semen sample were added to the right part (Well A). On the top of the dish, microdrops of Global Total with HEPES® medium were placed for subsequent ICSI. Additionally, two drops of Polyvinylpyrrolidone (PVP) with HSA—7% (Irvine Scientific, Fujifilm, Japan) were added to facilitate the control of the ICSI pipette. Subsequently, the dish was covered with approximately 4 mL of mineral oil (Lite® Oil, LifeGlobal, CooperSurgical, Denmark) and carefully placed in an incubator for 60 min at 37 °C.

### Intracytoplasmic sperm injection (ICSI)

After 60 min of incubation, the oocytes were carefully placed onto the dish for ICSI. Microinjection was performed on oocytes using spermatozoa selected either by cumulus cells (allocated to the study group lane) or exclusively by DGC (allocated to the control group lane), as determined by the randomization table. Following microinjection, the oocytes were transferred to culture dishes with microwells and subsequently incubated within the Geri® time-lapse system (Genea Biomedx, Australia). Embryonic culture conditions were maintained under controlled low oxygen pressure, ensuring stable temperature and humidity levels.

### Fertilization and embryo development

Fertilization was confirmed by the presence of two pronuclei and two polar bodies. Only properly fertilized oocytes were evaluated for embryo development on days 3 and 5 post ICSI. Following ICSI, embryos were cultured in a time-lapse incubation system under low oxygen and CO₂ pressure with controlled humidity, for a total duration of 5–6 days, using uninterrupted culture in Global Total® LP medium. Blastocysts were graded according to ASEBIR [16] considering blastocyst expansion, trophectoderm cell number at the equatorial plane, and the compactness of the inner cell mass. Embryo selection and categorization were conducted by senior embryologists without taking into account the experimental group allocation, ensuring unbiased evaluation.

### Statistical analysis

For categorical variables, a descriptive analysis was performed using frequency and percentage. For the univariate statistical analysis of comparison between study groups, the Chi-square test or Fisher’s exact test was used.

Numerical variables were presented as number of cases, mean, and standard deviation. For the evaluation of normal distributions, the Shapiro–Wilk test was performed. if the variable had a normal distribution, the univariate analysis was performed by Student’s *t*-test and otherwise by the Wilcoxon rank sum test.

In addition, a stratified analysis by maternal (26–35, 36–39, and 40–45) and paternal (20–30, 31–39, and 40–53) age ranges was performed. Age was not used as a covariate in each other group as there were no significant differences in the mean age of the groups.

Values of *p* < 0.05 were considered statistically significant.

Statistical analysis was performed with R statistical software, version 4.4.2 and Statistical Product and Service Solutions software, version 23.0 (SPSS, Chicago, IL, USA).

## Results

General variables were assessed between the control and study groups, as shown in Table 1. No significant differences were observed, indicating homogeneity. Depending on sperm motility, the number of spermatozoa penetrating the cumulus cells varied between cases; nevertheless, in all 99 cycles, an adequate number of spermatozoa was available to perform the ICSI procedure, and no cycles were excluded.

**Table 1:** General characteristics between study and control groups

Characteristic	COC^1^	Control^1^	***p***-value^2^
	*n* = 554	*n* = 543	
Female age	36.73 (3.78)	36.69 (3.80)	0.934
Male age	38.86 (4.73)	38.33 (4.47)	0.104
Seminal origin			1.0
Partner	554 (100.0%)	543.0 (100.0%)	
Sperm count (M/mL)	70.90 (50.35)	72.26 (49.06)	0.515
Motility (%)	58.41 (20.95)	58.55 (21.62)	0.863
Stimulation protocol			0.783
Antagonist	425 (76.7%)	408 (75.1%)	
Long agonist	48 (8.7%)	53 (9.8%)	
Progesterone-primed	81(14.6%)	82 (15.1%)	
Days of stimulation	10.43 (1.63)	10.57 (1.75)	0.100
Trigger			0.397
GnRH agonist	349 (63.8%)	328 (60.6%)	
hCG recombinant	63 (11.5%)	76 (14.0%)	
Mixed	135 (24.7%)	137 (25.3%)	
Cause of infertility			0.373
Unknown	61 (11.0%)	48 (8.8%)	
Female	332 (59.9%)	333 (61.3%)	
Male	65 (11.7%)	77 (14.2%)	
Mixed	96 (17.3%)	85 (15.7%)	

^1^Mean (SD); *n*/*N* (%)

^2^Wilcoxon rank sum test; Pearson’s chi-squared test; Fisher’s exact test

Across the 99 cycles, a total of 1387 oocytes were retrieved, of which 1097 (79.1%) reached metaphase II and were included in the oocyte-level allocation. In addition, the cumulus cells retrieved during oocyte aspiration were of adequate quality to be utilized in all cycles conducted throughout the study. In accordance with the primary objective of the study, blastocysts were classified as good quality (grades A + B) or lower quality (grades C + D) according to the ASEBIR criteria. Among blastocysts reaching the day-5 stage, the proportion of good-quality blastocysts was significantly higher in the study group compared with the control group (55.2% vs. 45.3%, *p* = 0.028) (Table 2).The fertilization outcomes showed no significant differences between the two groups (Table 3), indicating no differences in the number of oocytes that were correctly fertilized (2PN + 2 PB), those exhibiting anomalous fertilization (1PN and 3PN), or in the oocytes that degenerated following the microinjection process. The blastocyst formation rate was not statistically significant, although a trend favouring the study group was observed (62.8% vs. 58.3%, *p* = 0.178) (Table 3).

**Table 2:** Day 5 blastocyst quality among embryos reaching the blastocyst stage

	COC^1^ (***n*** = 250)	Control^1^ (***n*** = 245)	***p***-value^2^
Blastocyst quality			**0.028**
A_B	**138 (55.2%)**	**111 (45.3%)**	
C_D	**112 (44.8%)**	**134 (54.7%)**	

^1^*n* (%) (*n* = number of cases)

^2^Pearson’s chi-squared test

**Table 3:** Fertilization and blastocyst formation rates analysis between study and control groups (*PN* pronucleus, *PB* polar body, *DEG* degenerated, *NF* non-fertilized)

	COC^1^ (***n*** = 554)	Control^1^ (***n*** = 543)	***p***-value^2^
Fertilization (day 1)			**0.689**
2PN_2PB	**415 (74.9%)**	**424 (78.1%)**	
1PN_2PB	**17 (3.1%)**	**16 (2.9%)**	
3PN	**21 (3.8%)**	**18 (3.3%)**	
DEG	**31 (5.6%)**	**32 (5.9%)**	
NF_1PB	**67 (12.1%)**	**52 (9.6%)**	
NF_2PB	**3 (0.5%)**	**1 (0.2%)**	
Blastocyst formation rate (%)	**62.8**	**58.3**	**0.178**

^1^*n* (%) (*n* = number of cases)

^2^Pearson’s chi-squared test

In an exploratory subanalysis including embryos with complete time-lapse recordings, objective morphokinetic parameters with the greatest reported impact on embryo developmental competence were evaluated. No statistically significant differences were observed between the study and control groups in time to morula (TM: 84.47 ± 10.53 vs. 84.93 ± 10.20 h, *p* = 0.450) or time to blastocyst (TB: 108.88 ± 10.35 vs. 109.27 ± 10.45 h, *p* = 0.489), indicating no detectable modification of embryonic morphokinetic behaviour associated with cumulus cell–mediated sperm selection.

Based on the clinical data collected to date, the positive pregnancy rate and ongoing pregnancy rate were analyzed for both fresh embryo transfers and frozen embryo transfers (FET). The results, summarized in Table 4, revealed no statistically significant differences in the clinical parameters evaluated.

**Table 4:** Clinical outcomes between study and control groups

	COC^1^	Control^1^	***p***-value^2^
B-hCG (fresh)	**4.0/7.0 (57.1%)**	**1.0/4.0 (25.0%)**	**0.545**
Ongoing pregnancy (fresh)	**3.0/7.0 (42.9%)**	**1.0/4.0 (25.0%)**	** > 0.999**
B-hCG (FET)	**28.0/50.0 (56.0%)**	**17.0/31.0 (54.8%)**	**0.919**
Ongoing pregnancy (FET)	**22.0/48.0 (45.8%)**	**16.0/31.0 (51.6%)**	**0.616**

^1^*n*/*N* (%) (*n* = number of positive cases, *N* = number of total cases)

^2^Pearson’s chi-squared test

Additionally, an analysis stratified by maternal and paternal age ranges was conducted.

**Fig. 5:**
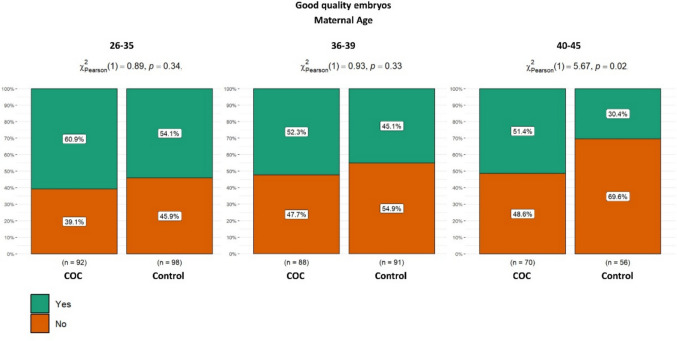
Proportion of good-quality day 5 blastocysts among evaluable blastocysts, stratified by maternal age

**Fig. 6:**
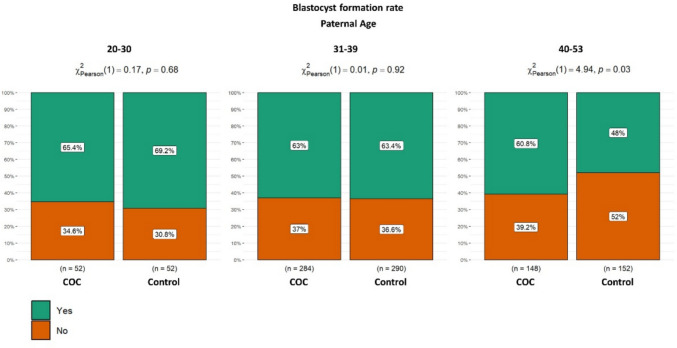
Blastocyst formation rate stratified by paternal age

Figure 5 shows the percentage of good-quality day 5 blastocysts (ASEBIR grades A + B) among blastocysts available for morphological assessment, stratified by maternal age (26–35, 36–39, and 40–45 years). No statistically significant differences were observed between the study and control groups in the 26–35 age group (60.9% vs. 54.1%, *p* = 0.344) or in the 36–39 age group (52.3% vs. 45.1%, *p* = 0.334). However, in women of advanced maternal age (40–45 years), a statistically significant improvement in day-5 blastocyst quality was observed, with the proportion of good-quality blastocysts increasing from 30.4% to 51.4% when cumulus cell–mediated sperm selection was applied (*p* = 0.017) (Fig. 5). No statistically significant differences in the rate of good-quality blastocysts were observed between the cumulus group and the control group across paternal age ranges. In the 20–30 age group, the proportions were similar between groups (58.3% vs. 61.8%, *p* = 0.770). Likewise, in the 31–39 age group, no significant difference was found (60.6% vs. 53.9%, *p* = 0.223). Although the proportion of good-quality blastocysts was higher in the cumulus group within the 40–53 age range (44.7% vs. 32.6%), the difference did not reach statistical significance (*p* = 0.083). Regarding one of the secondary objectives analyzed, blastocyst formation rates were significantly higher in the 40–53 age group in the cumulus group compared to the control group (60.8% vs. 48.0%, *p* = 0.026) (Fig. 6).

## Discussion

The aim of this study has been to assess the impact of cumulus cell-mediated sperm selection on the blastocyst quality using sibling oocytes in couples undergoing ICSI.

This clinical trial demonstrates that cumulus cell-mediated sperm selection can enhance blastocyst quality in these ICSI cycles, particularly in couples of advanced maternal ages. Although no statistically significant differences were observed in the secondary variables analyzed (fertilization, blastocyst formation, and pregnancy outcomes), the consistent improvement in blastocyst quality suggests that a more physiological approach to sperm selection may offer clinical benefits, especially in selected patient populations in ICSI cycles. Consistent with previous pilot data [12], this trial further supports the potential of cumulus cell-mediated sperm selection to improve blastocyst quality and aligns with the results reported by Wang et al. [10]. While fertilization, blastocyst formation, and pregnancy outcomes did not reach statistical significance, the improved embryo quality observed in the study group highlights the promising clinical potential of this approach in specific patient cohorts.

Expanding on the methodology of a previous study [12], in which a conventional ICSI dish with two channels of sperm medium was employed for cumulus cell selection, a novel dish (Oosafe® ICSI Dish with Sperm Selection Channels, CE mark pending, patent publication number EP4303299) was developed to address potential limitations related to sample displacement and to ensure optimal conditions for sperm placement and selection. In contrast to earlier approaches, where microinjections were performed directly within the selection dish, the new design incorporates specific dimensions and grooves in the plastic to provide a secure and reproducible working environment. This innovation, developed following the evaluation of two prototypes, was manufactured for use in the current study and received approval from the appropriate Institutional Review Board for Drug Research.

This study’s robust design is further reinforced by the randomization at the oocyte level, which ensures even distribution of sibling oocytes between the study and control groups, as highlighted in Table 1. Importantly, the homogeneity of the groups was confirmed through the absence of significant differences in general variables, which strengthens the reliability of our comparisons. In addition, the inclusion and exclusion criteria outlined in the ‘ Materials and methods’ section helped eliminate potential biases related to parental age. Notwithstanding, a subanalysis was conducted to explore the impact of parental age on the outcomes, ensuring the analysis remained robust and meaningful by selecting age ranges with sufficient sample sizes.

The findings of this clinical trial underline the promising potential of the new dish as a novel, physiologically based sperm selection method in ART. By mimicking the natural sperm selection process mediated by cumulus cells, this device offers several advantages over conventional methods. That is why this data, along with several other studies [9, 18, 19], suggest the selection of sperm with better DNA integrity, motility, and morphology, contributing to the significantly higher proportion of good-quality blastocysts observed in the study group. These results are consistent with the growing body of evidence supporting the beneficial role of cumulus cells in improving fertilization outcomes [9, 10].

The advantages of the *Oosafe® ICSI Dish with Sperm Selection Channels*, particularly in integrating sperm selection and microinjection into a single workflow, minimize the risk of iatrogenic damage while preserving the functional integrity of spermatozoa. By facilitating the use of cumulus cells in sperm selection, this method represents a significant advancement in ART. The natural secretome of cumulus cells contains progesterone and hyaluronic acid [6], which creates a microenvironment that supports sperm capacitation and hyperactivation, enhancing fertilization potential. Moreover, the extracellular matrix of cumulus cells serves as a physiological filter, favouring the selection of sperm with optimal genomic and structural characteristics. This advantage was particularly evident in older patient cohorts, where the device demonstrated improved blastocyst quality.

The standardized design of the dish enhances its reproducibility and facilitates consistent application in clinical practice. By ensuring consistent volume and placement of cumulus cells and sperm within designated wells, the dish reduces variability in sperm selection outcomes. The integration of a control lane allows for a direct comparison of sperm motility and quality with and without cumulus cell-mediated selection, thus strengthening the validity of the results and offering a robust framework for assessing the efficacy of this novel approach.

Despite the promising outcomes observed in this study, several limitations warrant consideration. The reliance on cumulus cells for sperm selection introduces logistical challenges, as the availability and quality of these cells are closely linked to the oocyte retrieval process. In patients with poor ovarian response or suboptimal cumulus–oocyte complexes, the feasibility of this approach may therefore be reduced. Moreover, its application requires specialized training for embryologists to ensure optimal use, which may increase the learning curve for widespread implementation in clinical practice.

In addition, the sample size calculation was based on theoretical assumptions regarding fertilization rates and losses during embryo culture, and the final number of oocytes included differed from this estimate. Accordingly, the primary outcome was analyzed among evaluable day 5 blastocysts derived from fertilized oocytes (2PN + 2 PB), assessing the proportion of good-quality embryos according to ASEBIR criteria. Although a statistically significant difference was observed for this endpoint, these considerations support a cautious interpretation of the findings, particularly with respect to extrapolation beyond embryo developmental outcomes.

Although blastocyst morphological grading represents a partially subjective outcome measure, the use of standardized ASEBIR criteria reflects routine clinical practice for embryo selection, transfer, and cryopreservation. To minimize subjectivity, embryo evaluation was performed by senior embryologists blinded to group allocation, and the sibling-oocyte study design further reduced inter-patient variability. Nevertheless, additional objective developmental endpoints, such as time-lapse morphokinetic parameters or oocyte utilization rates, were not included among the predefined outcomes of this study. In this context, an exploratory subanalysis including key morphokinetic parameters with major developmental relevance (time to morula and time to blastocyst) was performed in embryos with complete time-lapse recordings. No statistically significant differences were observed between study groups, indicating that cumulus cell-mediated sperm selection does not modify the overall kinetic behaviour of embryo development. These findings should therefore be interpreted primarily in the context of embryo developmental competence rather than definitive clinical impact.

Another limitation of this study is the absence of statistically significant differences in certain secondary outcomes, such as blastocyst formation rates and pregnancy outcomes across the overall study population. While trends favouring the use of cumulus cell-mediated sperm selection were observed, the results underscore the need for larger, multicenter studies to validate these findings and to assess long-term clinical benefits, including live birth rates and neonatal outcomes. Despite these limitations, the observed improvements in embryo quality suggest that the use of this dish could offer significant clinical benefits, particularly for certain patient groups. For example, in patients of advanced maternal or paternal age, this device presents a targeted strategy to counteract the negative effects of age-related declines in gamete quality. Moreover, its ability to select sperm with enhanced DNA integrity [13] may benefit patients with high levels of sperm DNA fragmentation, offering a more physiological alternative to current sperm selection methods that often fall short in addressing this issue.

The potential advantages of this innovative approach go beyond improving blastocyst quality. By mimicking the natural selection process of cumulus cells, the *Oosafe® ICSI Dish with Sperm Selection Channels* could enhance fertilization potential, thereby improving the overall success rates of ART procedures. As such, it offers the potential to reduce the need for more invasive or costly interventions, making it a highly relevant tool in the broader context of fertility treatments.

Looking ahead, further research is necessary to explore the full clinical implications of this technology. This includes investigating its effectiveness in cases of severe male factor infertility or in combination with other advanced sperm selection techniques, such as magnetic-activated cell sorting (MACS) or microfluidics. Comparative studies assessing the cost-effectiveness of the dish relative to conventional methods will also be essential to determine its broader applicability in routine clinical practice.

In conclusion, this device represents a promising advancement in assisted reproductive technologies, combining a physiologically based sperm selection approach with a streamlined laboratory workflow. In this sibling-embryo study, the use of cumulus cell–mediated sperm selection was associated with a significant improvement in day 5 blastocyst morphological quality. These findings suggest a potential benefit for embryo developmental competence under routine clinical conditions.

However, given the study design and sample size considerations, the results should be interpreted with appropriate caution and primarily in relation to embryo developmental outcomes. While the observed improvements in blastocyst quality may have clinical relevance, particularly in populations of advanced maternal or paternal age, the impact on downstream clinical outcomes such as implantation, pregnancy, and live birth rates remains to be established. Further adequately powered studies with longer follow-up are warranted to confirm these findings and to fully evaluate the clinical utility of the Oosafe® ICSI Dish with Sperm Selection Channels.

## Data Availability

The data supporting the findings of this study are not openly available due to concerns related to participant privacy and confidentiality. However, the datasets can be made available upon reasonable request, subject to appropriate review and oversight. Access will be granted under our supervision to ensure that all ethical and legal obligations regarding data protection are upheld.
